# Direct inhibition of PI3K in combination with dual HER2 inhibitors is required for optimal antitumor activity in HER2+ breast cancer cells

**DOI:** 10.1186/bcr3601

**Published:** 2014-01-23

**Authors:** Brent N Rexer, Siprachanh Chanthaphaychith, Kimberly Brown Dahlman, Carlos L Arteaga

**Affiliations:** 1 Division of Hematology-Oncology, Department of Medicine, School of Medicine, Vanderbilt University, 2220 Pierce Avenue, 777 PRB, Nashville, TN 37232-6307, USA; 2 Department of Cancer Biology, Vanderbilt University, 2220 Pierce Avenue, 777 PRB, Nashville, TN 37232-6307, USA; 3 Breast Cancer Research Program, Vanderbilt-Ingram Cancer Center, 2220 Pierce Avenue, 777 PRB, Nashville, TN 37232-6307, USA; 4 Vanderbilt-Ingram Cancer Center, Vanderbilt University, 2220 Pierce Avenue, 777 PRB, Nashville, TN 37232-6307, USA

## Abstract

**Introduction:**

Despite multiple advances in the treatment of HER2+ breast cancers, resistance develops even to combinations of HER2 targeting agents. Inhibition of PI3K pathway signaling is critical for the efficacy of HER2 inhibitors. Activating mutations in *PIK3CA* can overlap with *HER2* amplification and have been shown to confer resistance to HER2 inhibitors in preclinical studies.

**Methods:**

Lapatinib-resistant cells were profiled for mutations in the PI3K pathway with the SNaPshot assay. Hotspot *PIK3CA* mutations were retrovirally transduced into *HER2*-amplified cells. The impact of *PIK3CA* mutations on the effect of HER2 and PI3K inhibitors was assayed by immunoblot, proliferation and apoptosis assays. Uncoupling of PI3K signaling from HER2 was investigated by ELISA for phosphoproteins in the HER2-PI3K signaling cascade. The combination of HER2 inhibitors with PI3K inhibition was studied in HER2-amplified xenograft models with wild-type or mutant *PIK3CA*.

**Results:**

Here we describe the acquisition of a hotspot *PIK3CA* mutation in cells selected for resistance to the HER2 tyrosine kinase inhibitor lapatinib. We also show that the gain of function conferred by these *PIK3CA* mutations partially uncouples PI3K signaling from the HER2 receptor upstream. Drug resistance conferred by this uncoupling was overcome by blockade of PI3K with the pan-p110 inhibitor BKM120. In mice bearing *HER2*-amplified wild-type *PIK3CA* xenografts, dual HER2 targeting with trastuzumab and lapatinib resulted in tumor regression. The addition of a PI3K inhibitor further improved tumor regression and decreased tumor relapse after discontinuation of treatment. In a *PIK3CA*-mutant HER2+ xenograft, PI3K inhibition with BKM120 in combination with lapatinib and trastuzumab was required to achieve tumor regression.

**Conclusion:**

These results suggest that the combination of PI3K inhibition with dual HER2 blockade is necessary to circumvent the resistance to HER2 inhibitors conferred by *PIK3CA* mutation and also provides benefit to HER2+ tumors with wild-type *PIK3CA* tumors.

## Introduction

Amplification of the *HER2* oncogene occurs in approximately 25% of human breast cancers and predicts response to therapies targeting human epidermal growth factor receptor 2 (HER2), including trastuzumab, a monoclonal antibody directed against HER2, and lapatinib, a tyrosine kinase inhibitor (TKI) of HER2 and epidermal growth factor receptor (EGFR) [1,2]. HER2 is a member of the ErbB family of receptor tyrosine kinases (RTKs), which form both homo- and heterodimers, resulting in the activation of downstream signaling pathways [3]. In *HER2*-amplified cancers, the heterodimer of HER2 with kinase-deficient HER3 is a major activator of phosphoinositide 3-kinase (PI3K)-Akt signaling, and HER3, when phosphorylated, can directly couple to the p85 subunit of PI3K [4]. *HER2*-amplified tumors show significant reliance on PI3K-Akt signaling [5,6]. Importantly, inhibition of PI3K-Akt signaling is believed to be an essential component of the antitumor effect of HER2-directed therapies [7-9].

Alteration of the PI3K-Akt pathway is frequent in human cancers, and among the most frequent alterations are mutations in phosphoinositide 3-kinase catalytic subunit α (*PIK3CA*), the gene encoding the p110α catalytic subunit of PI3K. These mutations cluster in hotspot regions in the helical and kinase domains of p110α [10,11] and confer a gain of function [12]. *PIK3CA* hotspot mutations are found in approximately 25% of breast cancers and can overlap with *HER2* amplification [10,13,14]. The presence of these mutations in *HER2*-amplified cancer cells confers resistance to trastuzumab or lapatinib [7,15-17]. Moreover, aberrant activation of PI3K-Akt signaling by a *PIK3CA* mutation and/or phosphatase and tensin homologue (PTEN) loss is associated with resistance to trastuzumab in patients in some studies [15,18,19].

Recent clinical studies have suggested that targeting HER2-PI3K signaling with combinations of agents that inhibit HER2 by different mechanisms is more effective than a single HER2 inhibitor; combining trastuzumab and lapatinib was more effective than trastuzumab alone in both the metastatic and neoadjuvant settings [20,21]; and combining two HER2 antibodies, trastuzumab and pertuzumab, prolonged survival longer than trastuzumab alone [22]. Preclinical studies have suggested that the HER2/HER3 signaling complex has sufficient buffering capacity to withstand incomplete inhibition of HER2 catalytic activity, even in combination with a PI3K inhibitor, though this capacity can be overcome by fully inactivating HER2 catalytic activity with elevated doses of a TKI that may not be tolerated in clinical practice [23]. Moreover, even so-called dual-targeting of HER2 may not be sufficient to overcome resistance to HER2 inhibition, particularly in the case of *HER2*-amplified cancer with a *PIK3CA* mutation [16,24]. We have previously shown that, once resistance to HER2 inhibitors is established, inhibition of PI3K added to continued HER2 inhibition can overcome resistance [25].

In this work, we show that *PIK3CA*-activating mutations can be acquired during the development of resistance to HER2 inhibitors and that the presence of these mutations uncouples PI3K signaling from HER2. We further demonstrate that adding a PI3K inhibitor to dual-targeting of HER2 is more effective than HER2 targeting alone in a PI3K wild-type tumor and that the combination of HER2 and PI3K targeting is required for tumor regression in a model with *HER2* amplification and *PIK3CA* mutation.

## Methods

### Cell cultures, inhibitor treatments and proliferation and apoptosis assays

BT474, SKBR3, MDA-MB-361, HCC1954 and UACC893 cells were obtained from the American Type Culture Collection (Manassas, VA, USA). SUM190 cells were purchased from Asterand (Detroit, MI, USA). Lapatinib-resistant (LR) cell lines were generated as described previously [25] and cultured in the presence of 1 to 2 μM lapatinib. Lapatinib ditosylate and BIBW2992 were obtained from LC Laboratories (Woburn, MA, USA). BKM120 was obtained from Selleck Chemicals (Houston, TX, USA). Trastuzumab and pertuzumab were obtained from the Vanderbilt University Medical Center outpatient pharmacy. Unless otherwise noted, cells were treated with inhibitors at the following concentrations: lapatinib, 1 μM; trastuzumab, 10 μg/ml; BKM120, 1 μM; and BIBW2992, 1 μM. Cell proliferation was measured using the sulforhodamine B (SRB) reagent. Cells plated in 96-well plates were treated with inhibitors and fixed in 1% trichloroacetic acid after 72-hour treatment. Plates were rinsed with water and air-dried, then stained with 0.4% SRB in 1% acetic acid. Excess stain was removed by washing with 1% acetic acid, and plates were air-dried. Stained cells were solubilized in 10 mM Tris–HCl, pH 7.4, and absorbance at 590 nm was measured in a plate reader. Apoptosis was measured at 24 hours using the Caspase-Glo reagent (Promega, Madison, WI, USA) according to the manufacturer’s instructions. For longer-term growth assays, cells were seeded into six-well plates and treated with inhibitors as indicated. Media and inhibitors were replenished twice weekly, and cells were grown for 2 to 3 weeks until confluence in the untreated wells. Cells were fixed and stained in 20% methanol with 0.5% crystal violet and washed with water. Dried plates were imaged on a flatbed scanner.

### Generation of *PIK3CA* mutant cells

BT474 and SKBR3 parental cells were transduced with an amphotrophic retrovirus (LZRS) with neomycin resistance produced by Phoenix-AMPHO packaging cells containing C-terminal hemagglutinin (HA)-tagged bovine wild-type *PIK3CA* or E545K or H1047R mutations cloned from the JP1520 retroviral vector, as described previously [26]. To generate LR cell lines, transduced cells were treated with 1 μM lapatinib for approximately 4 to 6 weeks. Wild-type *PIK3CA*-expressing cells did not survive selection. Mutant-expressing, lapatinib-selected cells were maintained in the presence of 1 μM lapatinib.

### SNaPshot genotyping and Sanger sequencing

Genomic DNA was isolated from parental and LR BT474 cells and analyzed by the SNaPshot mutational profiling method (SNaPshot; Vanderbilt, NY, USA). This assay involves multiplexed polymerase chain reaction (PCR) and multiplexed single-base primer extension, followed by capillary electrophoresis [27,29]; Vandana G Abramson *et al*., unpublished data. The current assay was designed to detect 18 somatic point mutations in three genes (Additional file 1: Table S1). Briefly, PCR primers were pooled to amplify the target DNA, and PCR was performed using the following conditions: 95°C (8 minutes), followed by 40 cycles at (95°C (20 seconds), 58°C (30 seconds) and 72°C (1 minute)) and then a final extension at 72°C (3 minutes) (Additional file 2: Table S2). Next, PAGE-purified primers were pooled together, and multiplex single-base extension reactions were performed on ExoSAP-IT-treated (USB/Affymetrix, Santa Clara, CA, USA) PCR products using the following conditions: 96°C (30 seconds), followed by 35 cycles at (96°C (10 seconds), 50°C (5 seconds) and 60°C (30 seconds)) (Additional file 3: Table S3). Extension products were applied to capillary electrophoresis in an ABI 3730 DNA Analyzer (Applied Biosystems, Foster City, CA, USA), and the data were interpreted using ABI GeneMapper software (version 4.0; Applied Biosystems). Human male genomic DNA (Promega) was used as a wild-type control. Spiking primers were mixed to create a pan-positive control mix for the assay (Additional file 4: Table S4).

To validate the SNaPshot result, exon 9 of *PIK3CA* was amplified by high-fidelity PCR from genomic DNA and sequenced by traditional Sanger sequencing methods. The primer sequences are as follows: 5′-TTCAGCAGTGTGGTAAAGTTC-3′ forward; 5′GAGGCCAATCTTTTACCAAGC-3′ reverse.

### Immunoblot analysis and p85 immunoprecipitation

Cells were treated with inhibitors for 3 hours, and lysates were prepared as described previously [25]. Lysates were resolved on 7.5% acrylamide gels and transferred to Immobilon-FL PVDF (EMD Millipore, Billerica, MA, USA) and incubated in primary antibodies overnight at 4°C. Blots were washed and incubated with infrared fluorescent dye secondary antibody conjugates (LI-COR Biosciences, Lincoln, NE, USA), and blots were imaged using a LI-COR Odyssey scanner. Antibodies from the following sources were used for analysis: pHER2 Y1248 (R&D Systems, Minneapolis, MN, USA); Y877 pHER2 (Epitomics, Burlingame, CA, USA); Y1221/2 pHER2, Y11197 and Y1289 pHER3, S473 pAkt, Akt, S240/44 pS6, pErk1/2, Erk, and p110α PI3K (Cell Signaling Technology, Danvers, MA, USA); p85 N-terminal Src homology 2 (SH2) domain (EMD Millipore); HA (Covance, Princeton, NJ, USA); actin (Sigma-Aldrich, St Louis, MO, USA); HER2 (Thermo Fisher, Pittsburgh, PA, USA); and glyceraldehyde 3-phosphate dehydrogenase (Santa Cruz Biotechnology, Santa Cruz, CA, USA).

To analyze p110α isoforms bound to p85, lysates were prepared from wild-type or mutant-expressing cells and incubated with p85 rabbit antibody (directed against the N-terminal SH2 domain) overnight at 4°C as described previously [30]. Antibody complexes were isolated with Dynabeads Protein A (Life Technologies) and washed. Beads were boiled in SDS sample buffer and resolved on polyacrylamide gels, then immunoblotted with HA, p110α and p85 antibodies. Bands were quantitated using the LI-COR Odyssey scanner and Image Studio software. Mean values from triplicate experiments were compared by analysis of variance (ANOVA).

### Phosphoprotein ELISA analysis

PathScan Sandwich ELISA kits for pHER2, pAkt and pS6 were purchased from Cell Signaling Technology and used according to the manufacturer’s instructions. Lysates from cells treated with a range of lapatinib doses for 4 hours were prepared, and protein concentration determined by bicinchoninic acid assay (Pierce Biotechnology, Rockford, IL, USA). Lysates were diluted and incubated in enzyme-linked immunosorbent assay (ELISA) plates overnight at 4°C, then washed and developed according to the protocol. Absorbance values were read on a BioTek Epoch microplate optical reader (BioTek, Winooski, VT, USA) and normalized for each experiment to values for untreated cells. ELISA experiments were repeated in triplicate. Mean values ± SEM from the three experiments were used to plot log(inhibitor) vs. response curves using a variable slope model and to determine half-maximal inhibitory concentrations (IC_50_) using GraphPad Prism software (GraphPad Software, La Jolla, CA, USA). For statistical analysis, IC_50_ values for each phosphoprotein were compared between wild-type and mutant cells by ANOVA.

### Xenograft experiments

Animal studies were approved by the Vanderbilt University Medical Center Institutional Animal Care and Use Committee. BT-474 or HCC1954 cells (approximately 5 × 10^6^ in 50% Matrigel) were injected into female athymic nude mice. For BT474 cells, mice were implanted with 60-day, 0.72-mg, slow-release estrogen pellets (Innovative Research of America, Sarasota, FL, USA) on the day prior to injection. After tumors reached ≥250 mm^3^, mice were randomly assigned to treatment with trastuzumab (30 mg/kg by intraperitoneal injection twice weekly), lapatinib (100 mg/kg by oral gavage daily) and/or BKM120 (30 mg/kg by oral gavage daily). Tumors were measured with calipersn and tumor volume in cubic millimeters was calculated by the formula length/(2 × width^2^). Mean tumor volumes for each treatment group are displayed on log_2_ scale. Kaplan-Meier curves were constructed using the time point at which tumor volumes exceeded 100 mm^3^.

## Results

### Acquired *PIK3CA* mutation in lapatinib-resistant cells

**Figure 1:**
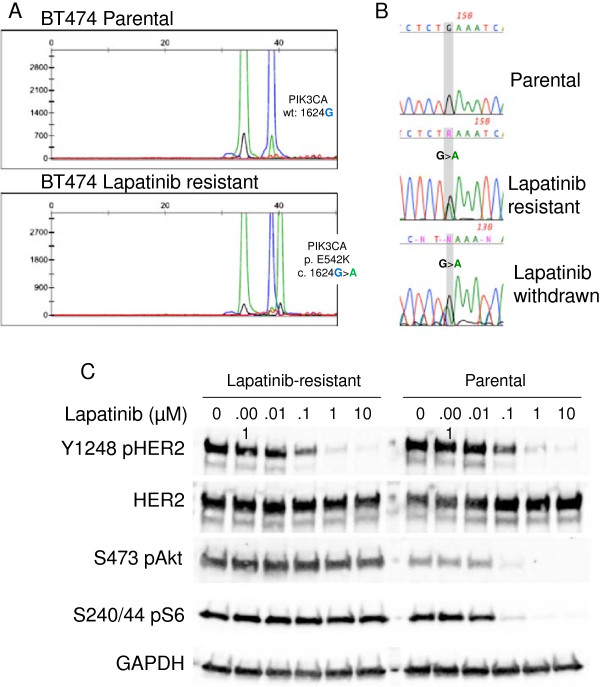
**BT474 lapatinib-resistant cells acquire E542K *PIK3CA *mutation. (A)** SNaPshot analysis of genomic DNA isolated from parental and lapatinib-resistant (LR) cells reveals a distinct peak corresponding to a G1624A nucleotide change in resistant cells. **(B)** Sanger sequencing of exon 9 of *PIK3CA* confirms the presence of the G1624A nucleotide change in LR cells. **(C)** Lysates from parental and LR cells treated with increasing doses of lapatinib, ranging from 0.001 to 10 μM, were analyzed by immunoblotting with the indicated antibodies.

We previously showed that LR cells exhibit continued activation of PI3K signaling despite inhibition of the HER2 tyrosine kinase [25]. To identify mechanisms associated with maintenance of PI3K signaling in these drug-resistant cells, we profiled them using the SNaPshot assay [27–29; Vandana G Abramson *et al*., unpublished] for a panel of mutations in *PIK3CA*, *AKT* and *PTEN* (Additional file 1: Table S1). Four of the six LR cell lines were derived from cells with preexisting hotspot *PIK3CA* mutations. None of the resistant cell lines acquired *AKT* or *PTEN* mutations. However, BT-474 LR cells acquired an E542K *PIK3CA* mutation (Figure 1A). We confirmed the presence of this mutation in cDNA by Sanger sequencing. Parental cells did not have any detectable E542K mutant sequence. The mutation persisted in LR cells even after culture in the absence of lapatinib for more than 2 weeks (Figure 1B). The E542 mutation is unique to BT474 cells. Other *HER2*-amplified cell lines known to contain *PIK3CA* mutations have either an E545K or H1047R mutation [10,14].

### *PIK3CA* mutation partially uncouples HER2 inhibition for phosphoinositide 3-kinase signaling

We observed that the four *HER2*-amplified cell lines that contain *de novo PIK3CA* mutations develop resistance to lapatinib more rapidly than *PIK3CA* wild-type cells in chronic cell culture. Thus, because the BT474 cells acquired a *PIK3CA* mutation upon the development of lapatinib resistance, we reasoned that the gain of function conferred by this mutation in p110α uncouples downstream PI3K signaling from HER2 and thereby blunts the inhibitory effect of lapatinib. We first evaluated response to lapatinib in parental and LR BT474 cells. Resistant cells were cultured in the absence of lapatinib for at least 2 weeks until recovery of HER2 phosphorylation was observed. After treatment of cells with increasing doses of lapatinib, we observed a similar inhibition of HER2 phosphorylation in parental and resistant cells, but the resistant cells with an acquired E542K mutation showed only very limited inhibition of PI3K signaling as measured by Akt and S6 phosphorylation (Figure 1C).

**Figure 2:**
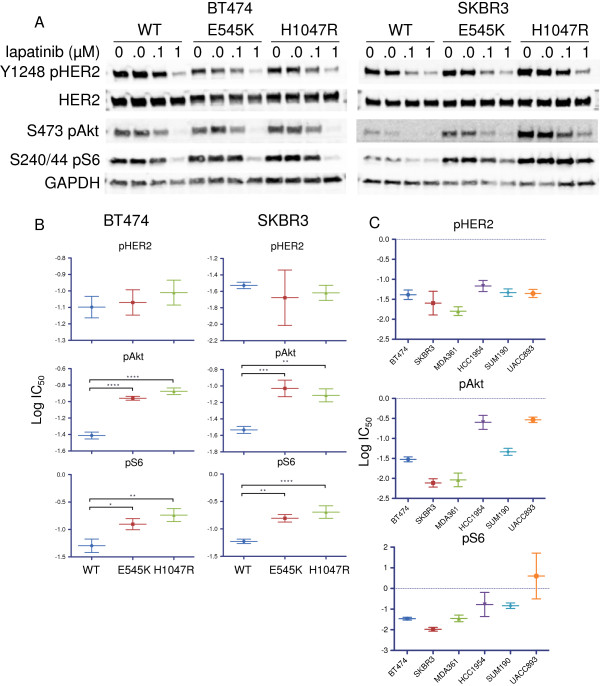
***PIK3CA *mutation uncouples phosphoinositide 3-kinase signaling from HER2 inhibition by lapatinib. (A)** BT474 and SKBR3 cells infected with wild-type, E545K or H1047R constructs were treated with lapatinib at the indicated doses, and lysates were analyzed by immunoblotting with the indicated antibodies. **(B)** Lysates from *PIK3CA* wild-type or mutant expressing cells treated with a range of lapatinib doses (0.0016 to 5 μM) were analyzed by ELISA for pHER2, pAkt and pS6. Half-maximal concentration (IC_50_) values were calculated, and the mean log IC_50_ ± SEM values for three replicate dose–inhibitor curves are shown. *P < 0.05, **P < 0.01, ***P < 0.001 and ****P < 0.0001. **(C)** HER2+ cell lines with wild-type *PIK3CA* (BT474 or SKBR3) or with a *PIK3CA* mutation (MDA361, HCC1954, SUM190 or UACC893) were treated with varying lapatinib doses and analyzed as described in **(B)**. Mean log IC_50_ values from three replicates ± SEM are shown. Mean IC_50_ data are shown in Table 1.

We next transduced parental BT474 and SKBR3 cells with wild-type, E545K or H1047R *PIK3CA* retroviral constructs. Expression of the ectopic p110α construct was verified by Western blot analysis for a C-terminal HA-tag (Additional file 5: Figure S1). We then evaluated the inhibition of pHER2 and PI3K signaling with a range of lapatinib doses. Similarly to BT474 LR cells with acquired E542K mutation, cells with the other hotspot mutations showed a blunted inhibitor response of PI3K signaling to lapatinib with persistent PI3K-Akt signaling (Figure 2A).

To better quantify the signaling output of mutant *PIK3CA* in cells in which HER2 was inhibited, we measured pHER2 by ELISA in cells treated with lapatinib and compared the IC_50_ for HER2 inhibition to the IC_50_ for PI3K inhibition as measured by Akt S473 and S6 S240/244 phosphorylation. We first compared BT474 and SKBR3 cells (without endogenous hotspot mutations) infected with wild-type, E545K or H1047R p110α retroviral constructs. As expected, IC_50_ data for inhibition of HER2 by lapatinib were similar between wild-type and *PIK3CA* mutant expressing cells (Figure 2B). However, both cell lines expressing either mutant *PIK3CA* isoform showed a significant increase in the IC_50_ for both Akt and S6 phosphorylation (Figure 2B and Additional file 6: Figure S2). For cells expressing ectopic mutant PI3K, the increase in IC_50_ was typically two- to threefold higher for the mutant cells than for the wild-type cells (Table 1). Interestingly, we observed a similar increase in the IC_50_ for pAkt and pS6 in the LR BT474 cells with an acquired E542K mutation (Additional file 7: Figure S3). These data suggest that mutant p110α uncouples PI3K signaling from HER2.

**Table 1:** **Mean half-maximal inhibitory concentrations for lapatinib inhibition of HER2 and signaling proteins in *PIK3CA *** **mutant cells**^**a**^

	**HER2**	**Akt**	**Increase over WT**	**S6**	**Increase over WT**
BT474	0.0411	0.0300		0.0347	
BT474 LR	0.0204	0.3925	13.1	0.7863	22.7
BT474 wt	0.0798	0.0387		0.0506	
BT474 E545K	0.0852	0.1098	2.8	0.1251	2.5
BT474 H1047R	0.0978	0.1336	3.4	0.1829	3.6
SKBR3	0.0254	0.0077		0.0106	
SKBR3 wt	0.0297	0.0293		0.0593	
SKBR3 E545K	0.0211	0.0936	3.2	0.1563	2.6
SKBR3 H1047R	0.0241	0.0770	2.6	0.2028	3.4
MDA361	0.0160	0.0092	0.5	0.0354	1.6
HCC1954	0.0681	0.2527	13.4	0.1678	7.4
SUM190	0.0465	0.0464	2.5	0.1461	6.5
UACC893	0.0444	0.2920	15.5	4.0040	177.0

^a^WT, Wild type. Half-maximal inhibitory concentration (IC_50_) values for inhibition of HER2, Akt and S6 phosphorylation by lapatinib treatment were determined by enzyme-linked immunosorbent assay (dose–response curves are shown in Additional file 6: Figure S2, Additional file 7: Figure S3 and Additional file 8: Figure S4). Mean IC_50_ values derived from at least three separate experiments are shown. For phosphoinositide 3-kinase signaling proteins Akt and S6, IC_50_ values from cells with *PIK3CA* mutations were compared to average values for cells without hotspot mutations (BT474 and SKBR3).

We next tested whether a similar partial uncoupling of PI3K signaling from HER2 activation was occurring in cells with endogenous PI3K hotspot mutations. We measured the IC_50_ values for HER2 and PI3K inhibition in four cell lines with endogenous *PIK3CA* mutations and compared them to the data for BT474 and SKBR3 cells. Again, we observed similar magnitudes of inhibition of HER2, but cell lines with endogenous PI3K mutations, particularly H1047R, showed an increased IC_50_ for pAkt and, in one cell line, for pS6 (Figure 2C and Additional file 8: Figure S4). There was a trend toward increased IC_50_ for inhibition of S6 phosphorylation by lapatinib, but the difference was not statistically significant. We observed in the dose–response curves that the level of the remaining S6 phosphorylation, even at maximal (5 μM) doses of lapatinib, was higher than that of wild-type cells (Additional file 8: Figure S4). This finding confirms that cell lines with endogenous PI3K mutations have a blunted inhibitory response and explains in part how resistance to lapatinib may emerge more quickly in *PIK3CA* mutant cells.

### Lapatinib-resistant cells preferentially utilize H1047R mutant *PIK3CA*

**Figure 3:**
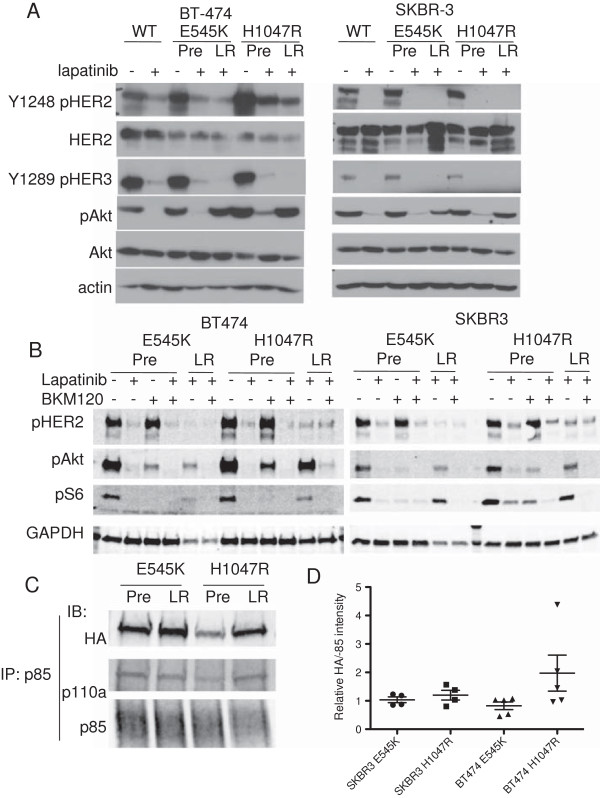
**Lapatinib-resistant *PIK3CA *mutant cell lines show reactivation of phosphoinositide 3-kinase and utilize mutant p110α for phosphoinositide 3-kinase signaling. (A)** Cells expressing E545K or H1047R were selected for lapatinib resistance. Unselected cells (Pre) were treated with lapatinib for 3 hours and analyzed along with lapatinib-resistant (LR) cells in the presence of lapatinib by immunoblotting with the indicated antibodies. **(B)** Lapatinib-sensitive and LR cells were treated with lapatinib and/or BKM120 for 3 hours, and lysates were analyzed by immunoblotting. **(C)** Phosphoinositide 3-kinase (PI3K) was immunoprecipitated from *PIK3CA* mutant–expressing cells before and after selection for lapatinib resistance with a p85 antibody. Complexes were separated by SDS-PAGE, and expression of mutant p110α was determined by immunoblot analysis for the hemagglutinin (HA)-tag. **(D)** HA band intensity detected by infrared fluorescence was quantified using a LI-COR Odyssey imaging system. Mean HA intensity normalized to p85 intensity from at least four replicate experiments is shown (bars = SEM).

We reasoned that cells that developed resistance to lapatinib by exploiting the uncoupling of PI3K from HER2 inhibition to allow escape from that inhibition would rely on the gain of function conferred by the mutant p110α isoform to allow for continued signaling in the presence of lapatinib. Thus, after selection for resistance, we hypothesized that the pool of p110α in complex with p85 and available for PI3K signaling would contain more mutant p110α than that available before selection for resistance. To test this hypothesis, BT474 and SKBR3 cells expressing ectopic HA-tagged E545K or H1047R p110α were selected for lapatinib resistance for 4 to 6 weeks. After an initial period of slower growth, the lapatinib-selected cells proliferated. We observed continued inhibition of HER2 by lapatinib, but recovery of Akt and S6 phosphorylation (Figure 3A). In LR cells, PI3K-Akt signaling was still dependent on p110α catalytic activity, as treatment with the PI3K inhibitor BKM120 abolished S473 Akt phosphorylation in both parental and LR ectopic *PIK3CA* mutant–expressing cells (Figure 3B).

We hypothesized that the gain of function conferred by the mutant p110α isoform in the LR cells would result in the preferential engagement of the mutant isoform for PI3K signaling under the selective pressure of HER2 inhibition. As an indirect measure of this utilization of mutant PI3K, we tested whether the PI3K signaling complex in the LR cells would be enriched for the mutant p110α isoform. We immunoprecipitated the pool of p110α in a complex with p85 using a p85 antibody and evaluated the level of mutant isoform present before and after lapatinib selection by immunoblotting for the HA-tag. In BT474 cells, we observed that more of the H1047R mutant p110α was detected in a complex with p85 in resistant cells than in parental cells (Figures 3C and 3D). We found a similar increase in HA expression in p85 immunoprecipitates from SKBR3 cells expressing H1047R p110α, but this increase did not persist after normalizing for levels of immunoprecipitated p85 (Figure 3D and Additional file 9: Figure S5). We did not observe any increase in levels of the E545K mutant in a complex with p85 in either cell line. The E545K mutation has been proposed to alter interaction with p85 and so may have a different mechanism of action than the H1047R catalytic site mutation [12,31]. This may explain why levels of E545K mutant p110α did not increase in PI3K from LR cells.

### HER2 inhibitor resistance conferred by phosphoinositide 3-kinase mutation is susceptible to p110α inhibition

**Figure 4:**
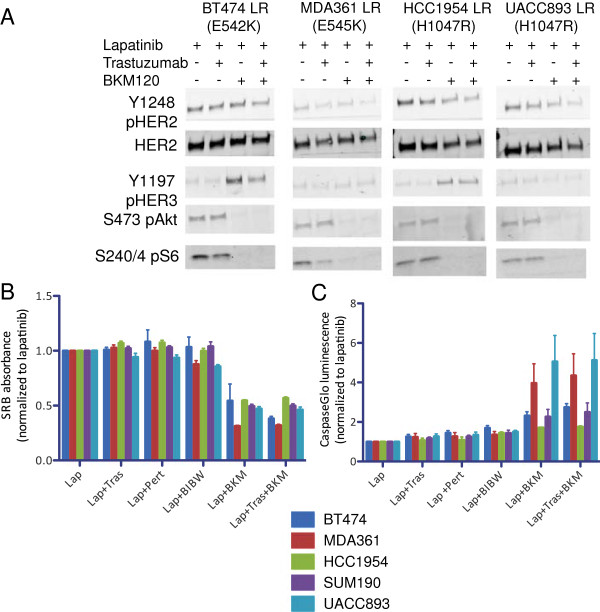
**Blockade of phosphoinositide 3-kinase, but not additional HER2 blockade, inhibits phosphoinositide 3-kinase signaling in lapatinib-resistant cells with *PIK3CA *mutation. (A)** Lapatinib-resistant cells in the presence of lapatinib were treated with trastuzumab overnight, with BKM120 or with the combinations as indicated, and lysates were analyzed by immunoblotting. **(B)** LR cell lines were plated in 96-well plates. Twenty-four hours after plating, cells were treated with inhibitors as indicated and fixed after seventy-two hours of treatment (lap = lapatinib, tras = trastuzumab, pert = pertuzumab and BKM = BKM120). Cells were stained with sulforhodamine B (SRB). Mean SRB absorbance normalized to cells treated with lapatinib only is shown (duplicates from two separate experiments; bars = SEM). **(C)** Cells were treated as described in **(B)**, and apoptosis was measured by luminescence after 24-hour treatment with the Caspase-Glo reagent. Mean luminescence (duplicates from two experiments) normalized to lapatinib only is displayed (bars = SEM).

The data we compiled in this study suggest that the gain of function conferred by *PIK3CA* mutations partially uncouples PI3K signaling from HER2 and allows for eventual escape from HER2 inhibition, but that signaling remains susceptible to inhibitors of p110α. This idea is in agreement with previous reports that lapatinib resistance from PI3K mutations or PTEN loss could be overcome by combining lapatinib with a dual PI3K/mammalian target of rapamycin (PI3K/mTOR) inhibitor [16]. Another possibility is that the gain of function of mutant PI3K can amplify low levels of HER2 signaling that remain after single-inhibitor treatment of HER2+ breast cancer [23], but the combination of dual HER2 inhibitor blockade may reduce this low level of signaling and thus inhibit even *PIK3CA* mutant cancers. This idea is consistent with recent clinical data indicating that the combination of trastuzumab and lapatinib or trastuzumab and pertuzumab is more effective than single HER2 inhibitor therapy [20-22]. To test whether additional HER2 blockade could overcome resistance to single-agent lapatinib or trastuzumab conferred by PI3K mutation, we treated LR *PIK3CA* mutant cells with lapatinib and trastuzumab and with or without the PI3K inhibitor BKM120. We found that the addition of trastuzumab did not inhibit the already low level of HER2 phosphorylation or further diminish the minimally detectable HER3 phosphorylation in these cells, nor did it further decrease PI3K-Akt signaling through Akt or S6 phosphorylation (Figure 4A). This low level of signaling appears to be sufficient for cell proliferation, as ectopic PI3K expression allowed for continued proliferation of cells in the presence of both trastuzumab and lapatinib (Additional file 10: Figure S6). Only the addition of the PI3K inhibitor resulted in PI3K pathway inhibition (Figure 4A). We also tested the addition of pertuzumab, a monoclonal antibody against HER2 that recognizes a different epitope than trastuzumab, and BIBW2992, a covalent inhibitor of EGFR/HER2 RTKs. None of these additional HER2 inhibitors resulted in any decrease in cell proliferation or increase in apoptosis in LR cells; only the addition of PI3K inhibition resulted in decreased proliferation and the induction of apoptosis (Figures 4B and 4C).

### HER2 inhibitors in combination with phosphoinositide 3-kinase inhibitors can prevent outgrowth of resistant tumors

**Figure 5:**
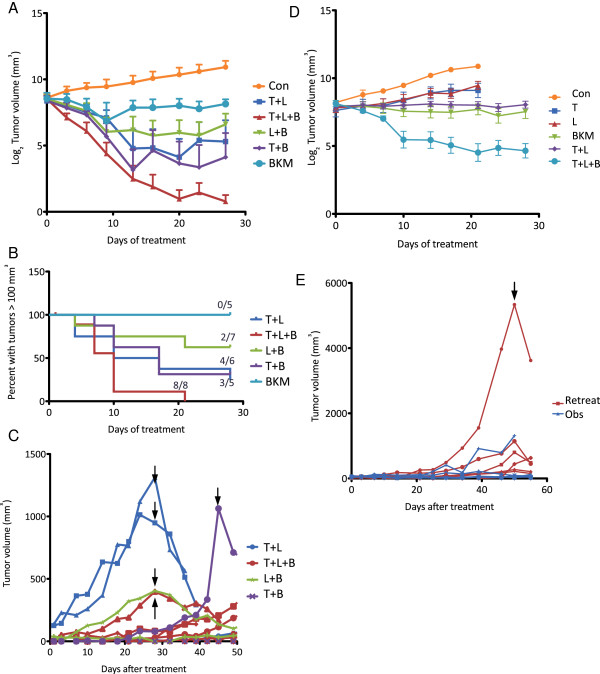
**Phosphoinositide 3-kinase inhibition combined with HER2 inhibition is more effective at inducing tumor regression than HER2 inhibition alone. (A)** Mice were injected with BT474 cells, and, after tumor formation, mice were treated with trastuzumab (T), lapatinib (L) or BKM120 (B) in the combinations indicated. Tumor growth was measured twice weekly. The log_2_ tumor volume data is shown over the 28-day treatment course (bars = SEM). **(B)** Kaplan-Meier plot showing the number of mice in each treatment group whose tumors regressed to below 100 mm^3^ in volume during treatment. The number of responses and the total number in each group are displayed above the curve. **(C)** After 28 days of treatment, mice whose tumors had regressed were followed for recurrence. Tumor volume for individual mice is plotted with the color according to the initial treatment group. Arrows indicate retreatment with T + L + B. **(D)** Mice were injected with HCC1954 cells (H1047R *PIK3CA* mutant), and, after tumor formation, mice were treated with trastuzumab (T), lapatinib (L) or BKM120 (B) alone or in the combinations indicated. Tumor growth was measured twice weekly. The log_2_ tumor volume data are shown over the 28-day treatment course (bars = SEM). Control, T and L alone mice were killed at day 21 of treatment because of excessive tumor volume. **(E)** After 28 days of treatment, mice from the T + L + B group whose tumors had regressed were followed for recurrence. Tumor volumes for individual mice are plotted. Red indicates mice whose tumors regrew, and those retreated with T + L + B are indicated by the arrow.

Because we did not find any benefit associated with additional HER2 inhibitors once resistance to an HER2 inhibitor was established in cell culture, we sought to test whether addition of a PI3K inhibitor to the combination of lapatinib and trastuzumab would prevent the outgrowth of resistant tumors, both with and without PI3K mutations. We first used BT474 cells (without PI3K hotspot mutations). As previously reported [32], trastuzumab and lapatinib together induced BT474 tumor regression. Treatment with BKM120 alone was able to block tumor growth, but did not induce any tumor regression. Combination of a single inhibitor of HER2 with a PI3K inhibitor appeared to be somewhat effective at inducing tumor regression, with a magnitude of benefit similar to the trastuzumab/lapatinib combination. Only the combination of all three drugs was sufficient to induce a robust regression in tumor growth, however, such that all of the mice in the treatment group showed complete regression of tumor growth after about 3 weeks of treatment (Figure 5B). At the end of 4 weeks of treatment, only residual tumors in the BKM120-only or dual-therapy groups were available for analysis, and we observed blockade of PI3K-Akt signaling by the PI3K inhibitor in these residual tumors (Additional file 11: Figure S7). Although the combination of all three drugs was better able to induce tumor regression than two-drug combinations, there were some mice in each group with complete tumor regression with dual- or triple-therapy. To determine whether the response to all three drugs was more durable than two-drug combinations, we followed mice after 28 days of treatment for tumor regrowth. Tumors recurred more rapidly in the trastuzumab/lapatinib combination–treated group, although all groups had at least one mouse with recurrent tumor growth. In all cases where significant recurrence was observed, retreatment with the combination of all three drugs appeared to be effective (black arrows in Figure 5C).

We next tested the combination of HER2 and PI3K inhibition in a xenograft model of HCC1954 cells with endogenous an H1047R *PIK3CA* mutation. These tumors showed continued growth in the presence of trastuzumab or lapatinib as single agents, further suggesting that *PIK3CA* mutations confer resistance to HER2 inhibitors (Figure 5D). In these tumors, the combination of trastuzumab and lapatinib was unable to induce tumor regression as it did in BT474 xenografts. BKM120 alone was as effective at inhibiting growth as dual HER2 inhibition, but the combination of HER2 and PI3K inhibitors was required to induce tumor regression. Unlike treatment of HER2+/*PIK3CA* wild-type tumors, no complete regressions were observed in any treatment group, though most tumors in the triple-therapy group remained only barely palpable at the end of treatment. After 28 days, treatment was stopped and mice were again followed for tumor recurrence, which eventually developed in about half of the mice (Figure 5E). Retreatment with all three inhibitors was able to reinduce tumor regression, suggesting that tumors remain sensitive to the combination of all three drugs. These results imply that a longer duration of treatment would be necessary to induce complete regression in tumors with the *PIK3CA* mutation.

## Discussion

The importance of the PI3K–Akt axis in oncogenic signaling is becoming increasingly apparent, especially in the case of HER2+ breast cancer, where inhibition of PI3K signaling is critical for the antitumor action of HER2 inhibitors and activating mutations in the PI3K pathway can confer resistance to HER2 inhibitors. We show in our present study that acquisition of a hotspot *PIK3CA* mutation is a mechanism of acquired resistance to lapatinib and that *PIK3CA* mutations partially uncouple PI3K from HER2 to allow for the development and maintenance of resistance. Further, targeting of PI3K itself, in combination with maximal HER2 blockade with both an antibody and a TKI, is more effective than HER2 targeting alone for HER2 tumors without *PIK3CA* mutations and is required for HER2 tumors with *PIK3CA* mutations.

We and others have found that both helical and catalytic domain mutations of *PIK3CA* can confer resistance to HER2 inhibitors [7,16,24,33]. In our biochemical assays, ectopic expression of either mutation appeared to uncouple HER2 inhibition from PI3K signaling to a similar degree (Figure 2), and cells expressing either mutation showed reactivation of PI3K upon the development of resistance [25]. When we assayed resistant cells for the proportion of mutant vs. wild-type p110α in the PI3K signaling complex compared to sensitive cells, however, we observed an increase in utilization of the H1047R mutant isoform but not the E545K isoform. We also did not observe the same degree of uncoupling of downstream signaling in a cell line with endogenous E545K compared with H1047R expressing cell lines (Figure 2). This is consistent with different proposed mechanisms for these mutations, whereby the helical domain mutant may function primarily to abolish the normal regulatory inhibition of PI3K, whereas the increased catalytic activity conferred by the kinase domain mutation may be required by the mutant cells still under the selective pressure of HER2 inhibition [12,31]. In either case, catalytic inhibition of p110α was effective at blocking downstream signaling for both mutations.

This catalytic inhibition of p110α is emerging as an attractive possible therapeutic option, with a number of inhibitors currently in preclinical and clinical development [34]. Several studies have investigated the potential importance of PI3K inhibition as a therapeutic strategy in *HER2*-amplified breast cancer, but these studies have uncovered feedback loops and other factors that may limit the use of PI3K inhibitors as single agents. A p110-specific inhibitor, GDC-0941, inhibited the growth of *HER2*-amplified cells in culture, but the combination of GDC-0941 with trastuzumab was required for tumor growth inhibition in mice [7]. Interestingly, the combination of GDC-0941 with antibody inhibitors of HER2 (trastuzumab and pertuzumab) appeared to be more effective than GDC-0941 in combination with a TKI [35]. In HER2+ cells with *PIK3CA* mutations, low doses of lapatinib are ineffective, but the cells are susceptible to dual PI3K/mTOR inhibitors such as BEZ235 [16] or INK-128 [36]. In the latter study, however, combination of the HER2 TKI with the PI3K inhibitor was not able to induce tumor regression, whereas combination of a PI3K p110 inhibitor (BKM120) with trastuzumab did result in tumor regression, albeit in a wild-type *PIK3CA* model. In a transgenic HER2+/*PIK3CA* H1047R mutant mouse model, tumors that were resistant to the combinations of trastuzumab and lapatinib or trastuzumab and pertuzumab could be inhibited by BKM120 alone or in combination with HER2 inhibitors [24]. Those observations are in agreement with our present findings that BKM120 alone did not induce tumor regression, but did result in tumor regression when combined with HER2 inhibitors. This increased efficacy of HER2 and PI3K inhibitor combinations may be partly explained by increased Erk signaling and feedback upregulation of HER3 after PI3K inhibitor treatment alone, whereas targeting HER2 in combination with PI3K inhibition could overcome these compensatory mechanisms [37,38].

Our results support the clinical testing of combinations of PI3K inhibition with maximal HER2 inhibition for HER2+ breast cancer. In biomarker studies from the recent CLEOPATRA clinical trial, although the combination of trastuzumab and pertuzumab was superior to trastuzumab alone, regardless of *PIK3CA* mutation status, the magnitude of benefit was less for those tumors with *PIK3CA* mutations than for wild-type tumors [39]. There is a potential for overlapping toxicities when combining multiple inhibitors, so important questions regarding selection of patients and the sequence and timing of combination therapies remain to be addressed. Our data suggest that *PIK3CA* mutation can be acquired during HER2 inhibitor treatment and that the presence of a *PIK3CA* mutation requires PI3K blockade in addition to HER2 blockade. Despite several lines of preclinical evidence that either *PIK3CA* mutation can confer HER2 inhibitor resistance, a robust correlation of the occurrence of *PIK3CA* mutations with outcomes after trastuzumab or lapatinib therapy in patients is still lacking. A recent study of PTEN expression and *PIK3CA* mutation in HER2+ patients with either recurrent disease after trastuzumab treatment or progression of metastatic disease while on trastuzumab therapy showed a significantly increased frequency of either PTEN loss or *PIK3CA* mutation compared to untreated HER2+ tumors, but *PIK3CA* mutation status alone did not appear to be significantly enriched in the clinically trastuzumab-refractory cohort [19]. In addition to determining whether *PIK3CA* mutation predicts for the eventual development of resistance, there is a critical need to understand how frequently *PIK3CA* mutations occur after patients develop resistance to HER2 inhibitor treatment. Another important question is whether these combinations need to be given together as part of the initial treatment or whether direct PI3K inhibition can be added after therapeutic resistance develops. In all of our models of resistance, we found reactivation of PI3K signaling and susceptibility to PI3K inhibition. We also found that additional HER2 blockade after the development of lapatinib resistance was ineffective and that PI3K inhibition was essential to overcoming resistance. This suggests that a potentially better-tolerated sequential approach might be effective, and, indeed, sequential additive treatment with HER2 inhibitors appears to have some clinical efficacy, though these patients eventually develop resistance to combined HER2 blockade [21]. In our xenograft models, however, we found that up-front combination of both HER2 and PI3K blockade was most effective at inducing regression and preventing tumor regrowth, regardless of PI3K mutation status. In the case of *PIK3CA* mutation, this combination was required to induce tumor regression. This suggests that the combination of HER2 and PI3K therapies up front, if tolerable, might provide optimal treatment for patients with *PIK3CA* mutations and also have potential benefits for patients with *PIK3CA* wild-type tumors.

## Conclusions

Our results show that the gain of function conferred by *PIK3CA* mutations contributes to resistance to HER2 inhibitors by partially uncoupling PI3K signaling from HER2 inhibition. They also show that, though maximal HER2 blockade is insufficient to overcome the effects of the mutation, addition of a PI3K inhibitor to HER2 inhibitors can reverse or prevent resistance. This suggests that this combination may be an effective strategy to overcome resistance in patients that warrants clinical testing.

## Abbreviations

ANOVA: Analysis of variance; EGFR: Epidermal growth factor receptor; ELISA: Enzyme-linked immunosorbent assay; HER2: Human epidermal growth factor receptor 2; HER3: Human epidermal growth factor receptor 3; IC50: Half-maximal inhibitory concentration; LR: Lapatinib-resistant; PCR: Polymerase chain reaction; PI3K: Phosphoinositide 3-kinase; PIK3CA: Phosphoinositide 3-kinase catalytic subunit α; RTK: Receptor tyrosine kinase; SRB: sulforhodamine B; TKI: Tyrosine kinase inhibitor.

## Competing interests

The authors declare that they have no conflicts of interest.

## Authors’ contributions

BR and CLA conceived and designed experiments. BR and SC performed experiments and analyzed data. KD contributed the SNapShot assay and acquired and interpreted the SNaPshot data. BR and CLA drafted the manuscript. All authors read and approved the final version of the manuscript.

## Supplementary Material

Additional file 1: Table S1 The breast cancer SNaPshot screen queries 18 point mutations in 3 genes Click here for file

Additional file 2: Table S2 Breast cancer SNaPshot screen multiplex polymerase chain reaction primers Click here for file

Additional file 3: Table S3 Single-base extension primers for the breast cancer SNaPshot screen Click here for file

Additional file 4: Table S4 Breast SNaPshot screen spiking primers used for pan-positive control assay Click here for file

Additional file 5: Figure S1 Expression of ectopic *PIK3CA* constructs. Lysates from parental cells or cells infected with wild-type, E545K (EK) or H1047R (HR) *PIK3CA* retroviral constructs were analyzed by immunoblotting with the indicated antibodies. The *PIK3CA* constructs contain a C-terminal hemagglutinin (HA)-tag Click here for file

Additional file 6: Figure S2 Phosphoinositide 3-kinase (PI3K) mutation shifts the half-maximal inhibitory concentration for lapatinib inhibition of PI3K signaling. Cells expressing wild-type or mutant PI3K constructs were treated with a range of lapatinib concentrations (0.0016 to 5 μM) and pHER2, pAkt and pS6 measured by enzyme-linked immunosorbent assay. Results were normalized to untreated cells and inhibitor response curves were fitted with GraphPad from a mean of three experiments (bars = SEM). Click here for file

Additional file 7: Figure S3 Acquired E542K mutation in BT474 lapatinib resistant cells shifts the lapatinib half-maximal inhibitory concentration (IC_50_) for phosphoinositide 3-kinase signaling. BT474 parental or lapatinib-resistant cells cultured without lapatinib for at least 2 weeks were treated with a range of lapatinib doses and analyzed by enzyme-linked immunosorbent assay for pHER2, pAkt and pS6. The inhibitor response curves and mean IC_50_ values from three separate experiments are displayed (bars = SEM) Click here for file

Additional file 8: Figure S4 Cell lines with endogenous phosphoinositide 3-kinase (PI3K) mutations show uncoupling of PI3K signaling from HER2 inhibition by lapatinib. **(A)** Cell lines (wild-type or with PI3K mutations as indicated) were treated with a range of lapatinib doses and analyzed by enzyme-linked immunosorbent assay for pHER2, pAkt and pS6. The inhibitor response curves derived from a mean of three separate experiments are shown. **(B)** The level of pS6 remaining at the 5 μM lapatinib dose, normalized to untreated cells, is shown (mean ± SEM) Click here for file

Additional file 9: Figure S5 Lapatinib-resistant cells utilize H1047R mutant *PIK3CA* for phosphoinositide 3-kinase (PI3K) signaling. Cells infected with E545K or H1047R mutant *PIK3CA* vectors were selected for lapatinib resistance. PI3K was immunoprecipitated from cells before and after selection for resistance, and the level of hemagglutinin (HA) expression was determined by immunoblot analysis of the immune complexes. HA band intensity was quantitated using infrared fluorescence secondary antibodies and LI-COR software. The relative intensity of HA expression in resistant cells compared with their unselected counterparts is shown. The mean value (±SEM) of five or six replicate immunoprecipitations is displayed Click here for file

Additional file 10: Figure S6 Phosphoinositide 3-kinase (PI3K) mutation allows for emergence of resistant colonies, even to dual HER2 blockade. Cells expressing wild-type or PI3K mutations as indicated were seeded into 12-well plates and were treated with lapatinib, trastuzumab or a combination of the two 24 hours after plating as indicated. Cells were grown for 2 to 3 weeks in media, and drugs were replenished twice weekly. At the end of treatment, the cells were fixed and stained with crystal violet Click here for file

Additional file 11: Figure S7 BKM120 is required to maximally inhibit phosphoinositide 3-kinase signaling in tumor xenografts. Tumors from BT474 cells that remained after 28 days of treatment with BKM120 (B), lapatinib (L) or trastuzumab (T) in the combinations indicated were harvested, and lysates were prepared and analyzed by immunoblotting with the indicated antibodies Click here for file

## References

[B1] Geyer CE Forster J Lindquist D Chan S Romieu CG Pienkowski T Jagiello-Gruszfeld A Crown J Chan A Kaufman B Skarlos D Campone M Davidson N Berger M Oliva C Rubin SD Stein S Cameron D Lapatinib plus capecitabine for HER2-positive advanced breast cancer N Engl J Med 2006 355 2733 2743 10.1056/NEJMoa064320 17192538

[B2] Slamon DJ Leyland-Jones B Shak S Fuchs H Paton V Bajamonde A Fleming T Eiermann W Wolter J Pegram M Baselga J Norton L Use of chemotherapy plus a monoclonal antibody against HER2 for metastatic breast cancer that overexpresses HER2 N Engl J Med 2001 344 783 792 10.1056/NEJM200103153441101 11248153

[B3] Yarden Y Sliwkowski MX Untangling the ErbB signalling network Nat Rev Mol Cell Biol 2001 2 127 137 10.1038/35052073 11252954

[B4] Holbro T Beerli RR Maurer F Koziczak M Barbas CF 3rd Hynes NE The ErbB2/ErbB3 heterodimer functions as an oncogenic unit: ErbB2 requires ErbB3 to drive breast tumor cell proliferation Proc Natl Acad Sci USA 2003 100 8933 8938 10.1073/pnas.1537685100 12853564 PMC166416

[B5] Brachmann SM Hofmann I Schnell C Fritsch C Wee S Lane H Wang S Garcia-Echeverria C Maira SM Specific apoptosis induction by the dual PI3K/mTor inhibitor NVP-BEZ235 in HER2 amplified and PIK3CA mutant breast cancer cells Proc Natl Acad Sci USA 2009 106 22299 22304 10.1073/pnas.0905152106 20007781 PMC2799764

[B6] O’Brien C Wallin JJ Sampath D GuhaThakurta D Savage H Punnoose EA Guan J Berry L Prior WW Amler LC Belvin M Friedman LS Lackner MR Predictive biomarkers of sensitivity to the phosphatidylinositol 3′ kinase inhibitor GDC-0941 in breast cancer preclinical models Clin Cancer Res 2010 16 3670 3683 10.1158/1078-0432.CCR-09-2828 20453058

[B7] Junttila TT Akita RW Parsons K Fields C Lewis Phillips GD Friedman LS Sampath D Sliwkowski MX Ligand-independent HER2/HER3/PI3K complex is disrupted by trastuzumab and is effectively inhibited by the PI3K inhibitor GDC-0941 Cancer Cell 2009 15 429 440 10.1016/j.ccr.2009.03.020 19411071

[B8] Ritter CA Perez-Torres M Rinehart C Guix M Dugger T Engelman JA Arteaga CL Human breast cancer cells selected for resistance to trastuzumab *in vivo* overexpress epidermal growth factor receptor and ErbB ligands and remain dependent on the ErbB receptor network Clin Cancer Res 2007 13 4909 4919 10.1158/1078-0432.CCR-07-0701 17699871

[B9] Yakes FM Chinratanalab W Ritter CA King W Seelig S Arteaga CL Herceptin-induced inhibition of phosphatidylinositol-3 kinase and Akt Is required for antibody-mediated effects on p27, cyclin D1, and antitumor action Cancer Res 2002 62 4132 4141 12124352

[B10] Saal LH Holm K Maurer M Memeo L Su T Wang X Yu JS Malmström PO Mansukhani M Enoksson J Hibshoosh H Borg A Parsons R *PIK3CA* mutations correlate with hormone receptors, node metastasis, and ERBB2, and are mutually exclusive with PTEN loss in human breast carcinoma Cancer Res 2005 65 2554 2559 10.1158/0008-5472-CAN-04-3913 15805248

[B11] Samuels Y Wang Z Bardelli A Silliman N Ptak J Szabo S Yan H Gazdar A Powell SM Riggins GJ Willson JK Markowitz S Kinzler KW Vogelstein B Velculescu VE High frequency of mutations of the *PIK3CA* gene in human cancers Science 2004 304 554 10.1126/science.1096502 15016963

[B12] Zhao L Vogt PK Helical domain and kinase domain mutations in p110α of phosphatidylinositol 3-kinase induce gain of function by different mechanisms Proc Natl Acad Sci USA 2008 105 2652 2657 10.1073/pnas.0712169105 18268322 PMC2268191

[B13] Koboldt DC Fulton RS McLellan MD Schmidt H Kalicki-Veizer J McMichael JF Fulton LL Dooling DJ Ding L Mardis ER Wilson RK Ally A Balasundaram M Butterfield YS Carlsen R Carter C Chu A Chuah E Chun HJ Coope RJ Dhalla N Guin R Hirst C Hirst M Holt RA Lee D Li HI Mayo M Moore RA Mungall AJ Cancer Genome Atlas Network Comprehensive molecular portraits of human breast tumours Nature 2012 490 61 70 10.1038/nature11412 23000897 PMC3465532

[B14] Stemke-Hale K Gonzalez-Angulo AM Lluch A Neve RM Kuo WL Davies M Carey M Hu Z Guan Y Sahin A Symmans WF Pusztai L Nolden LK Horlings H Berns K Hung MC van de Vijver MJ Valero V Gray JW Bernards R Mills GB Hennessy BT An integrative genomic and proteomic analysis of PIK3CA, PTEN, and AKT mutations in breast cancer Cancer Res 2008 68 6084 6091 10.1158/0008-5472.CAN-07-6854 18676830 PMC2680495

[B15] Berns K Horlings HM Hennessy BT Madiredjo M Hijmans EM Beelen K Linn SC Gonzalez-Angulo AM Stemke-Hale K Hauptmann M Beijersbergen RL Mills GB van de Vijver MJ Bernards R A functional genetic approach identifies the PI3K pathway as a major determinant of trastuzumab resistance in breast cancer Cancer Cell 2007 12 395 402 10.1016/j.ccr.2007.08.030 17936563

[B16] Eichhorn PJA Gili M Scaltriti M Serra V Guzman M Nijkamp W Beijersbergen RL Valero V Seoane J Bernards R Baselga J Phosphatidylinositol 3-kinase hyperactivation results in lapatinib resistance that is reversed by the mTOR/phosphatidylinositol 3-kinase inhibitor NVP-BEZ235 Cancer Res 2008 68 9221 9230 10.1158/0008-5472.CAN-08-1740 19010894 PMC2587064

[B17] Serra V Markman B Scaltriti M Eichhorn PJA Valero V Guzman M Botero ML Llonch E Atzori F Di Cosimo S Maira M Garcia-Echeverria C Parra JL Arribas J Baselga J NVP-BEZ235, a dual PI3K/mTOR inhibitor, prevents PI3K signaling and inhibits the growth of cancer cells with activating PI3K mutations Cancer Res 2008 68 8022 8030 10.1158/0008-5472.CAN-08-1385 18829560

[B18] Nagata Y Lan KH Zhou X Tan M Esteva FJ Sahin AA Klos KS Li P Monia BP Nguyen NT Hortobagyi GN Hung MC Yu D PTEN activation contributes to tumor inhibition by trastuzumab, and loss of PTEN predicts trastuzumab resistance in patients Cancer Cell 2004 6 117 127 10.1016/j.ccr.2004.06.022 15324695

[B19] Chandarlapaty S Sakr RA Giri D Patil S Heguy A Morrow M Modi S Norton L Rosen N Hudis C King TA Frequent mutational activation of the PI3K-AKT pathway in trastuzumab-resistant breast cancer Clin Cancer Res 2012 18 6784 6791 10.1158/1078-0432.CCR-12-1785 23092874 PMC3525734

[B20] Baselga J Bradbury I Eidtmann H Di Cosimo S de Azambuja E Aura C Gómez H Dinh P Fauria K Van Dooren V Aktan G Goldhirsch A Chang TW Horváth Z Coccia-Portugal M Domont J Tseng LM Kunz G Sohn JH Semiglazov V Lerzo G Palacova M Probachai V Pusztai L Untch M Gelber RD Piccart-Gebhart M NeoALTTO Study Team Lapatinib with trastuzumab for HER2-positive early breast cancer (NeoALTTO): a randomised, open-label, multicentre, phase 3 trial Lancet 2012 379 633 640 10.1016/S0140-6736(11)61847-3 22257673 PMC5705192

[B21] Blackwell KL Burstein HJ Storniolo AM Rugo HS Sledge G Aktan G Ellis C Florance A Vukelja S Bischoff J Baselga J O’Shaughnessy J Overall survival benefit with lapatinib in combination with trastuzumab for patients with human epidermal growth factor receptor 2–positive metastatic breast cancer: final results from the EGF104900 Study J Clin Oncol 2012 30 2585 2592 10.1200/JCO.2011.35.6725 22689807

[B22] Baselga J Cortés J Kim SB Im SA Hegg R Im YH Roman L Pedrini JL Pienkowski T Knott A Clark E Benyunes MC Ross G Swain SM CLEOPATRA Study Group Pertuzumab plus trastuzumab plus docetaxel for metastatic breast cancer N Engl J Med 2012 366 109 119 10.1056/NEJMoa1113216 22149875 PMC5705202

[B23] Amin DN Sergina N Ahuja D McMahon M Blair JA Wang D Hann B Koch KM Shokat KM Moasser MM Resiliency and vulnerability in the HER2-HER3 tumorigenic driver Sci Transl Med 2010 2 16ra7 20371474 10.1126/scitranslmed.3000389 PMC3033659

[B24] Hanker AB Pfefferle AD Balko JM Kuba MG Young CD Sánchez V Sutton CR Cheng H Perou CM Zhao JJ Cook RS Arteaga CL Mutant *PIK3CA* accelerates HER2-driven transgenic mammary tumors and induces resistance to combinations of anti-HER2 therapies Proc Natl Acad Sci USA 2013 110 14372 14377 10.1073/pnas.1303204110 23940356 PMC3761610

[B25] Rexer BN Ham AJL Rinehart C Hill S de Matos Granja-Ingram N González-Angulo AM Mills GB Dave B Chang JC Liebler DC Arteaga CL Phosphoproteomic mass spectrometry profiling links Src family kinases to escape from HER2 tyrosine kinase inhibition Oncogene 2011 30 4163 4174 10.1038/onc.2011.130 21499296 PMC3204390

[B26] Isakoff SJ Engelman JA Irie HY Luo J Brachmann SM Pearline RV Cantley LC Brugge JS Breast cancer–associated *PIK3CA* mutations are oncogenic in mammary epithelial cells Cancer Res 2005 65 10992 11000 10.1158/0008-5472.CAN-05-2612 16322248

[B27] Dias-Santagata D Akhavanfard S David SS Vernovsky K Kuhlmann G Boisvert SL Stubbs H McDermott U Settleman J Kwak EL Rapid targeted mutational analysis of human tumours: a clinical platform to guide personalized cancer medicine EMBO Mol Med 2010 2 146 158 10.1002/emmm.201000070 20432502 PMC3377316

[B28] Su Z Dias-Santagata D Duke M Hutchinson K Lin YL Borger DR Chung CH Massion PP Vnencak-Jones CL Iafrate AJ Pao W A platform for rapid detection of multiple oncogenic mutations with relevance to targeted therapy in non-small-cell lung cancer J Mol Diagn 2011 13 74 84 10.1016/j.jmoldx.2010.11.010 21227397 PMC3070558

[B29] Lovly CM Dahlman KB Fohn LE Su Z Dias-Santagata D Hicks DJ Hucks D Berry E Terry C Duke M Su Y Sobolik-Delmaire T Richmond A Kelley MC Vnencak-Jones CL Iafrate AJ Sosman J Pao W Routine multiplex mutational profiling of melanomas enables enrollment in genotype-driven therapeutic trials PLoS One 2012 7 e35309 10.1371/journal.pone.0035309 22536370 PMC3335021

[B30] Engelman JA Jänne PA Mermel C Pearlberg J Mukohara T Fleet C Cichowski K Johnson BE Cantley LC ErbB-3 mediates phosphoinositide 3-kinase activity in gefitinib-sensitive non-small cell lung cancer cell lines Proc Natl Acad Sci USA 2005 102 3788 3793 10.1073/pnas.0409773102 15731348 PMC553328

[B31] Huang CH Mandelker D Schmidt-Kittler O Samuels Y Velculescu VE Kinzler KW Vogelstein B Gabelli SB Amzel LM The structure of a human p110α/p85α complex elucidates the effects of oncogenic PI3Kα mutations Science 2007 318 1744 1748 10.1126/science.1150799 18079394

[B32] Garrett JT Olivares MG Rinehart C Granja-Ingram ND Sánchez V Chakrabarty A Dave B Cook RS Pao W McKinely E Manning HC Chang J Arteaga CL Transcriptional and posttranslational up-regulation of HER3 (ErbB3) compensates for inhibition of the HER2 tyrosine kinase Proc Natl Acad Sci USA 2011 108 5021 5026 10.1073/pnas.1016140108 21385943 PMC3064360

[B33] Chakrabarty A Rexer BN Wang SE Cook RS Engelman JA Arteaga CL H1047R phosphatidylinositol 3-kinase mutant enhances HER2-mediated transformation by heregulin production and activation of HER3 Oncogene 2010 29 5193 5203 10.1038/onc.2010.257 20581867 PMC2945381

[B34] Miller TW Rexer BN Garrett JT Arteaga CL Mutations in the phosphatidylinositol 3-kinase pathway: role in tumor progression and therapeutic implications in breast cancer Breast Cancer Res 2011 13 224 10.1186/bcr3039 22114931 PMC3315683

[B35] Yao E Zhou W Lee-Hoeflich ST Truong T Haverty PM Eastham-Anderson J Lewin-Koh N Gunter B Belvin M Murray LJ Friedman LS Sliwkowski MX Hoeflich KP Suppression of HER2/HER3-mediated growth of breast cancer cells with combinations of GDC-0941 PI3K inhibitor, trastuzumab, and pertuzumab Clin Cancer Res 2009 15 4147 4156 10.1158/1078-0432.CCR-08-2814 19509167

[B36] García-García C Ibrahim YH Serra V Calvo MT Guzmán M Grueso J Aura C Pérez J Jessen K Liu Y Rommel C Tabernero J Baselga J Scaltriti M Dual mTORC1/2 and HER2 blockade results in antitumor activity in preclinical models of breast cancer resistant to anti-HER2 therapy Clin Cancer Res 2012 18 2603 2612 10.1158/1078-0432.CCR-11-2750 22407832

[B37] Chakrabarty A Sánchez V Kuba MG Rinehart C Arteaga CL Feedback upregulation of HER3 (ErbB3) expression and activity attenuates antitumor effect of PI3K inhibitors Proc Natl Acad Sci USA 2012 109 2718 2723 10.1073/pnas.1018001108 21368164 PMC3286932

[B38] Serra V Scaltriti M Prudkin L Eichhorn PJ Ibrahim YH Chandarlapaty S Markman B Rodriguez O Guzman M Rodriguez S Gili M Russillo M Parra JL Singh S Arribas J Rosen N Baselga J PI3K inhibition results in enhanced HER signaling and acquired ERK dependency in HER2-overexpressing breast cancer Oncogene 2011 30 2547 2557 10.1038/onc.2010.626 21278786 PMC3107390

[B39] Baselga J Cortés J Im SA Kiermaier A Ross G Swain SM Biomarker analyses in CLEOPATRA: a phase III, placebo-controlled study of pertuzumab in HER2-positive, first-line metastatic breast cancer (MBC) Cancer Res 2012 72 Abstract nr S5-1 10.1200/JCO.2013.54.5384 25332247

