# Supplementary material for: Direct inhibition of PI3K in combination with dual HER2 inhibitors is required for optimal antitumor activity in HER2+ breast cancer cells
Source: Breast Cancer Res. 2014 Jan 23;16(1):R9. doi: 10.1186/bcr3601 (PMC3978602; doi:10.1186/bcr3601)
Supplement: Additional file 1: Table S1 — The breast cancer SNaPshot screen queries 18 point mutations in 3 genes [file bcr3601-S1.docx]

SUPPLEMENTAL TABLE 1. The breast cancer SNaPshot screen queries 18 point mutations in 3 genes.

| ***PIK3CA*** |  |  |
| --- | --- | --- |
| **Position** | **Amino acid mutant** | **Nucleotide mutant** |
| H1047 | p.H1047R | c.3140A>G |
|  | p.H1047L | c.3140A>T |
| E542 | p.E542K | c.1624G>A |
| E545 | p.E545K | c.1633G>A |
|  | p.E545Q | c.1633G>C |
|  | p.E545A | c.1634A>C |
|  | p.E545G | c.1634A>G |
|  | p.E545V | c.1634A>T |
| Q546 | p.Q546K | c.1636C>A |
|  | p.Q546E | c.1636C>G |
|  | p.Q546P | c.1637A>C |
|  | p.Q546R | c.1637A>G |
|  | p.Q546L | c.1637A>T |
| D549 | p.D549N | c.1645G>A |
|  |  |  |
| ***PTEN*** |  |  |
| R233 | p.R233^#^ | c.697C>T |
| R159 | p.R159S | c.477G>T |
| R267 | p.R267fs*9 | c.800delA |
|  |  |  |
| ***AKT*** |  |  |
| E17 | p.E17K | c.49G>A |
|  |  |  |

^#^This mutation results in a premature stop codon.

*This mutation results in a deletion-frameshift.
