# Supplementary material for: Direct inhibition of PI3K in combination with dual HER2 inhibitors is required for optimal antitumor activity in HER2+ breast cancer cells
Source: Breast Cancer Res. 2014 Jan 23;16(1):R9. doi: 10.1186/bcr3601 (PMC3978602; doi:10.1186/bcr3601)
Supplement: Additional file 2: Table S2 — Breast cancer SNaPshot screen multiplex polymerase chain reaction primers [file bcr3601-S2.docx]

SUPPLEMENTAL TABLE 2. Breast cancer SNaPshot screen multiplex-PCR primers.

| **Amplification primer name** | **Primer sequence^a^** | **Product length (bp)** |
| --- | --- | --- |
| AKT1_ex2_a1^b^ | GAGGGTCTGACGGGTAGAGT | 95 |
| AKT1_ex2_a2^b^ | TCTTGAGGAGGAAGTAGCGT |
| PIK3CA_ex9_a1^b^ | GACAAAGAACAGCTCAAAGCAA | 98 |
| PIK3CA_ex9_a2^b^ | TTTAGCACTTACCTGTGACTCCA |
| PIK3CA_ex20_a1^b^ | GAGCAAGAGGCTTTGGAGTA | 80 |
| PIK3CA_ex20_a2^b^ | ATCCAATCCATTTTTGTTGTCC |
| PTEN_ex5_b1 | GGGCAAATTTTTAAAGGCACAA | 106 |
| PTEN_ex5_b2 | CCAGGAAGAGGAAAGGAAAAA |
| PTEN_ex7_a1^b^ | GGTGAAGATATATTCCTCCAATTCA | 123 |
| PTEN_ex7_a2^b^ | TTCTCCCAATGAAAGTAAAGTACAAA |

^a^The sequences are shown 5’>3’.

^b^Primer sequences were published previously .
