# Supplementary material for: Direct inhibition of PI3K in combination with dual HER2 inhibitors is required for optimal antitumor activity in HER2+ breast cancer cells
Source: Breast Cancer Res. 2014 Jan 23;16(1):R9. doi: 10.1186/bcr3601 (PMC3978602; doi:10.1186/bcr3601)
Supplement: Additional file 3: Table S3 — Single-base extension primers for the breast cancer SNaPshot screen [file bcr3601-S3.docx]

SUPPLEMENTAL TABLE 3. Single-base extension primers for the breast cancer SNaPshot screen.

| **Extension primer name^a^** | **Primer sequence^b^** | **Primer length (nucleotides)** |
| --- | --- | --- |
| AKT149_extR^c^ | **ACTGACTGACTGACTGACTGACTGACTGACTGACTGACTGACTGACTGACT**CGCCAGGTCTTGATGTACT | 70 |
| PIK3CA1633_extF | **GACTGACTGACTGACTGACTGACTGACT**GATCCTCTCTCTGAAATCACT | 49 |
| PIK3CA1624_extF | **GACTGACTGACTGAC**CACGAGATCCTCTCTCT | 32 |
| PIK3CA1636_extF | **GACTGACTGACTGACTGACTGACTGACTGACTGACTGACTGACT**CCTCTCTCTGAAATCACTGAG | 65 |
| PIK3CA1637_extF | **GACTGACTGACTGACTGACTGACTGACTGACTGACTGAC**CTCTCTCTGAAATCACTGAGC | 60 |
| PIK3CA3140_extR | GTCCAGCCACCATGA | 15 |
| PIK3CA1645_extR | **GACTGACTGACTGACTGACTGACTGACTGACTGACTGACTGACTGACTGACT**CCTGTGACTCCATAGAAAAT | 72 |
| PIK3CA1634_extF | CTCTCTCTGAAATCACTG | 18 |
| PTEN477_extF | **GACTGACTGACTGACTGACTGACTGACTGACTGACTGACTGACTGACTGACTGACTGACTGACT**GATTTCTATGGGGAAGTAAG | 84 |
| PTEN697_extF | **ACTGACTGACTGACTGACTGACTGACTGACTGACTGA**CCAATTCAGGACCCACA | 54 |
| PTEN800delA_extF | **GACTGACTGACTGACTGACTGACTGACTGACTGACTGACT**AAACAGAACAAGATGCTAAAAA | 62 |

^a^Primers were purified by polyacrylamide gel electrophoresis.

^b^The sequences are shown 5’>3’ and bold nucleotides are repetitive GACT sequence used to adjust product size.

^c^Primer sequences were published previously [28].
