# Supplementary material for: Direct inhibition of PI3K in combination with dual HER2 inhibitors is required for optimal antitumor activity in HER2+ breast cancer cells
Source: Breast Cancer Res. 2014 Jan 23;16(1):R9. doi: 10.1186/bcr3601 (PMC3978602; doi:10.1186/bcr3601)
Supplement: Additional file 4: Table S4 — Breast SNaPshot screen spiking primers used for pan-positive control assay [file bcr3601-S4.docx]

SUPPLEMENTAL TABLE 4. Breast SNaPshot screen spiking primers used for pan-positive control assay.

| **Spiking primer name** | **Primer sequence^a^** |
| --- | --- |
| PIK3CA1633G>A^b^ | CTCCTGCTTAGTGATTTCAGAGAGAGGATCAAAAA |
| PIK3CA1633G>C^b^ | CTCCTGCTGAGTGATTTCAGAGAGAGGATCAAAAA |
| PTEN477G>T | GTCTCTGGTACTTACTTCCCCATAGAAATCAAAAA |
| PIK3CA1634A>C | TCTCCTGCGCAGTGATTTCAGAGAGAGGATAAAA |
| PIK3CA1634A>G | TCTCCTGCCCAGTGATTTCAGAGAGAGGATAAAA |
| PIK3CA1634A>T | TCTCCTGCACAGTGATTTCAGAGAGAGGATAAAA |
| PTEN800A(G) | GTACAAACCTTTTTAGCATCTTGTTCTGTAAAAA |
| AKT49G>A^b^ | CTGTAGGGAAGTACATCAAGACCTGGCGGCAAAAA |
| PIK3CA1624G>A | AGTGATTTTAGAGAGAGGATCTCGTGTAGAAAAA |
| PIK3CA3140A>G | ATGATGCACGTCATGGTGGCTGGACAACAATTTTT |
| PIK3CA3140A>T | ATGATGCACTTCATGGTGGCTGGACAACAATTTTT |
| PTEN697C>T | TTCCCGTCATGTGGGTCCTGAATTGGAGGAATAAAA |
| PIK3CA1637A>C | CTTTCTCCGGCTCAGTGATTTCAGAGAGAGAAA |
| PIK3CA1637A>G | CTTTCTCCCGCTCAGTGATTTCAGAGAGAGAAA |
| PIK3CA1637A>T | CTTTCTCCAGCTCAGTGATTTCAGAGAGAGAAA |
| PIK3CA1645G>A | CAGGAGAAAAATTTTCTATGGAGTCACAGAAAAA |
| PIK3CA1636C>A | CTTTCTCCTTCTCAGTGATTTCAGAGAGAGAAA |
| PIK3CA1636C>G | CTTTCTCCTCCTCAGTGATTTCAGAGAGAGAAA |

^a^The sequences are shown 5’>3’

^b^Primer sequences were published previously [28].
