# Supplementary material for: Direct inhibition of PI3K in combination with dual HER2 inhibitors is required for optimal antitumor activity in HER2+ breast cancer cells
Source: Breast Cancer Res. 2014 Jan 23;16(1):R9. doi: 10.1186/bcr3601 (PMC3978602; doi:10.1186/bcr3601)
Supplement: Additional file 5: Figure S1 — Expression of ectopic PIK3CA constructs. Lysates from parental cells or cells infected with wild-type, E545K (EK) or H1047R (HR) PIK3CA retroviral constructs were analyzed by immunoblotting with the indicated antibodies. The PIK3CA constructs contain a C-terminal hemagglutinin (HA)-tag [file bcr3601-S5.docx]

Supplemental Figure 1. Expression of ectopic *PIK3CA* constructs. Lysates from parental cells or cells infected with wild-type, E545K (EK), or H1047R (HR) *PIK3CA* retroviral constructs were analyzed by immunoblot with the indicated antibodies. The *PIK3CA* constructs contain a C-terminal HA tag.
