# Supplementary material for: Direct inhibition of PI3K in combination with dual HER2 inhibitors is required for optimal antitumor activity in HER2+ breast cancer cells
Source: Breast Cancer Res. 2014 Jan 23;16(1):R9. doi: 10.1186/bcr3601 (PMC3978602; doi:10.1186/bcr3601)
Supplement: Additional file 6: Figure S2 — Phosphoinositide 3-kinase (PI3K) mutation shifts the half-maximal inhibitory concentration for lapatinib inhibition of PI3K signaling. Cells expressing wild-type or mutant PI3K constructs were treated with a range of lapatinib concentrations (0.0016 to 5 μM) and pHER2, pAkt and pS6 measured by enzyme-linked immunosorbent assay. Results were normalized to untreated cells and inhibitor response curves were fitted with GraphPad from a mean of three experiments (bars = SEM). [file bcr3601-S6.docx]

Supplemental Figure 2. PI3K mutation shifts the IC_50_ for lapatinib inhibition of PI3K signaling. Cells expressing wild-type or mutant PI3K constructs were treated with a range of lapatinib concentrations (0.0016 – 5 µM) and pHER2, pAkt, and pS6 measured by ELISA. Results were normalized to untreated cells and inhibitor response curves were fitted with GraphPad from a mean of 3 experiments (bars=SEM).
