# Supplementary material for: Direct inhibition of PI3K in combination with dual HER2 inhibitors is required for optimal antitumor activity in HER2+ breast cancer cells
Source: Breast Cancer Res. 2014 Jan 23;16(1):R9. doi: 10.1186/bcr3601 (PMC3978602; doi:10.1186/bcr3601)
Supplement: Additional file 7: Figure S3 — Acquired E542K mutation in BT474 lapatinib resistant cells shifts the lapatinib half-maximal inhibitory concentration (IC50) for phosphoinositide 3-kinase signaling. BT474 parental or lapatinib-resistant cells cultured without lapatinib for at least 2 weeks were treated with a range of lapatinib doses and analyzed by enzyme-linked immunosorbent assay for pHER2, pAkt and pS6. The inhibitor response curves and mean IC50 values from three separate experiments are displayed (bars = SEM) [file bcr3601-S7.docx]

Supplemental Figure 3. Acquired E542K mutation in BT474 lapatinib resistant cells shifts the lapatinib IC_50_ for PI3K signaling. BT474 parental or lapatinib resistant cells cultured without lapatinib for at least 2 weeks were treated with a range of lapatinib doses and analyzed by ELISA for pHER2, pAkt, and pS6. The inhibitor response curves and mean IC_50_s from three separate experiments are displayed (bars=SEM).
