# Supplementary material for: Direct inhibition of PI3K in combination with dual HER2 inhibitors is required for optimal antitumor activity in HER2+ breast cancer cells
Source: Breast Cancer Res. 2014 Jan 23;16(1):R9. doi: 10.1186/bcr3601 (PMC3978602; doi:10.1186/bcr3601)
Supplement: Additional file 8: Figure S4 — Cell lines with endogenous phosphoinositide 3-kinase (PI3K) mutations show uncoupling of PI3K signaling from HER2 inhibition by lapatinib. (A) Cell lines (wild-type or with PI3K mutations as indicated) were treated with a range of lapatinib doses and analyzed by enzyme-linked immunosorbent assay for pHER2, pAkt and pS6. The inhibitor response curves derived from a mean of three separate experiments are shown. (B) The level of pS6 remaining at the 5 μM lapatinib dose, normalized to untreated cells, is shown (mean ± SEM) [file bcr3601-S8.docx]

Supplemental Figure 4. Cell lines with endogenous PI3K mutations show uncoupling of PI3K signaling from HER2 inhibition by lapatinib. A. Cell lines wild-type or with PI3K mutations as indicated were treated with a range of lapatinib doses and analyzed by ELISA for pHER2, pAkt, and pS6. The inhibitor response curves from a mean of three separate experiments is shown. B. The level of pS6 remaining at the 5 µM lapatinib dose, normalized to untreated cells, is also shown (mean +/- SEM).
