# Supplementary material for: Direct inhibition of PI3K in combination with dual HER2 inhibitors is required for optimal antitumor activity in HER2+ breast cancer cells
Source: Breast Cancer Res. 2014 Jan 23;16(1):R9. doi: 10.1186/bcr3601 (PMC3978602; doi:10.1186/bcr3601)
Supplement: Additional file 9: Figure S5 — Lapatinib-resistant cells utilize H1047R mutant PIK3CA for phosphoinositide 3-kinase (PI3K) signaling. Cells infected with E545K or H1047R mutant PIK3CA vectors were selected for lapatinib resistance. PI3K was immunoprecipitated from cells before and after selection for resistance, and the level of hemagglutinin (HA) expression was determined by immunoblot analysis of the immune complexes. HA band intensity was quantitated using infrared fluorescence secondary antibodies and LI-COR software. The relative intensity of HA expression in resistant cells compared with their unselected counterparts is shown. The mean value (±SEM) of five or six replicate immunoprecipitations is displayed [file bcr3601-S9.docx]

Supplemental Figure 5. Lapatinib-resistant cells utilize H1047R mutant *PIK3CA* for PI3K signaling. Cells infected with E545K or H1047R mutant *PIK3CA* vectors were selected for lapatinib resistance. PI3K was immunoprecipitated from cells before and after selection for resistance and the level of HA expression determined by immunoblot of the immune complexes. HA band intensity was quantitated using infrared fluorescent secondary antibodies and LiCor software. The relative intensity of HA expression in resistant cells compared with their unselected counterparts is shown and the mean value of 5-6 replicate immunoprecipitations is displayed +/- SEM.
