# Supplementary material for: Direct inhibition of PI3K in combination with dual HER2 inhibitors is required for optimal antitumor activity in HER2+ breast cancer cells
Source: Breast Cancer Res. 2014 Jan 23;16(1):R9. doi: 10.1186/bcr3601 (PMC3978602; doi:10.1186/bcr3601)
Supplement: Additional file 10: Figure S6 — Phosphoinositide 3-kinase (PI3K) mutation allows for emergence of resistant colonies, even to dual HER2 blockade. Cells expressing wild-type or PI3K mutations as indicated were seeded into 12-well plates and were treated with lapatinib, trastuzumab or a combination of the two 24 hours after plating as indicated. Cells were grown for 2 to 3 weeks in media, and drugs were replenished twice weekly. At the end of treatment, the cells were fixed and stained with crystal violet [file bcr3601-S10.docx]

Supplemental Figure 6. PI3K mutation allows for emergence of resistant colonies even to dual HER2 blockade. Cells expressing wild-type or PI3K mutations as indicated were seeded in 12-well plates and 24 h after plating treated with lapatinib, trastuzumab, or the combination as indicated. Cells were grown for 2-3 weeks with media and drugs replenished twice weekly. At the end of treatment cells were fixed and stained with crystal violet.
