# Supplementary material for: Direct inhibition of PI3K in combination with dual HER2 inhibitors is required for optimal antitumor activity in HER2+ breast cancer cells
Source: Breast Cancer Res. 2014 Jan 23;16(1):R9. doi: 10.1186/bcr3601 (PMC3978602; doi:10.1186/bcr3601)
Supplement: Additional file 11: Figure S7 — BKM120 is required to maximally inhibit phosphoinositide 3-kinase signaling in tumor xenografts. Tumors from BT474 cells that remained after 28 days of treatment with BKM120 (B), lapatinib (L) or trastuzumab (T) in the combinations indicated were harvested, and lysates were prepared and analyzed by immunoblotting with the indicated antibodies [file bcr3601-S11.docx]

Supplemental Figure 7. BKM120 is required to maximally inhibit PI3K signaling in tumor xenografts. Tumors from BT474 cells that remained after 28 days of treatment with BKM120 (B), lapatinib (L), or trastuzumab (T) in the combinations indicated were harvested and lysates were prepared and analyzed by immunoblot with the indicated antibodies.
